# Sickle Cell Disease and the Respiratory System: A Tangential Perspective to the Hematopulmonological Dilemma

**DOI:** 10.7759/cureus.15562

**Published:** 2021-06-10

**Authors:** Ibrahim Sange, Phani Bhavana Cherukuri, Vaishnavi Parchuri, Natasha Srinivas, Sruthi Priyavadhana Ramanan, Aliya H Sange, Srimy Modi, Farhat A Khot

**Affiliations:** 1 Research, California Institute of Behavioral Neurosciences & Psychology, Fairfield, USA; 2 Research, K. J. Somaiya Medical College, Mumbai, IND; 3 Research, Guntur Medical College, Guntur, IND; 4 Research, NRI Medical College, Guntur, IND; 5 Research, B.G.S. Global Institute of Medical Sciences, Bangalore, IND; 6 Neurology, California Institute of Behavioral Neurosciences & Psychology, California, USA; 7 Medicine/Surgery, Saveetha Medical College, Chennai, IND; 8 Research, Dubai Medical College, Dubai, ARE; 9 Research, Maharashtra Institute of Medical Education and Research (MIMER) Medical College, Pune, IND

**Keywords:** sickle cell disease, sickle cell anemia, acute chest syndrome, pulmonary hypertension, asthma

## Abstract

Sickle cell disease (SCD) is a genetically inherited hematological condition that predominantly affects the African-American subset of the population. It leads to the precipitation of multi-systematic manifestations throughout the course of the life of the patient leading to an increased rate of inpatient admissions and decreased quality of life. This article has reviewed some of the most common pulmonary complications of SCD with a brief overview of the clinical features and their management and has also highlighted the fatality of the complications placing a strong focus on screening, monitoring, and the treatment of the disease. The article has also discussed the management of SCD from a pulmonological perspective rather than hematological alone.

## Introduction and background

Sickle cell disease (SCD) is a group of heterogeneous hematological disorders that occur due to a genetic mutation in the beta-globin chain of hemoglobin leading to the sickling of the biconcave red blood cells (RBCs) and thereby hampering the movement of the RBCs through the circulation [1]. Although being relatively common in the people of Mediterranean, Asian, Caribbean, and Middle-Eastern descent, this disorder exhibits a generous inclination towards the African population and it results in a large number of deaths as an enormous fraction of the population with the disease go undiagnosed [1,2]. It is theorized that the prevalence of SCD in African populations is a defensive evolutionary mechanism to combat endemic diseases like malaria as the parasite thrives inside healthy RBCs but cannot survive for long in deformed RBCs [3]. SCD is an autosomal recessive disease that arises generally due to a point mutation in the sixth position of the beta-globin chain leading to the replacement of glutamic acid by valine [4]. The presence of the latter compound makes the hemoglobin chain vulnerable to undergoing polymerization and thereby sickling of the biconcave RBCs [4]. Genetically, there are two forms of the disease - heterozygous and homozygous. The heterozygous form, also known as the sickle cell trait, results in the presence of low amounts of sickled hemoglobin (HbS) compared to the homozygous form and the patients do not develop a severe form of the disease having a healthier course of life [5]. It is also imperative to appreciate the fact that the presence of fetal hemoglobin (HbF) in the fetal blood serves as a protective factor against the sickling of RBCs as it is capable of retaining more oxygen and hence, newborn babies usually do not develop clinical features at birth [6]. The homozygous variant results in the clinical precipitation of the gravest form of the disease resulting in a large spectrum of symptoms like dactylitis, acute chest syndrome, splenic sequestration crisis, strokes, etc., whenever the patient is subjected to any form of stress like infections, dehydration, and acidosis [1]. Although the laboratory picture is that of hemolytic anemia and sickled RBCs can be seen on a peripheral smear, the diagnosis is established by performing hemoglobin electrophoresis where the HbS hemoglobin being more positively charged than adult hemoglobin (HbA) demarcates itself thereby giving a positive test [7]. The management of the condition is multi-tangential and is dictated by the clinical presentation of the disease. Hydroxyurea, a drug that increases the concentration of HbF in the blood, has served as the mainstay of the treatment for a very long time [6]. Other treatment modalities like blood transfusions, folate supplementation, hydration, etc., are also included depending upon the acuity and severity of the disease [6]. The involvement of the lungs in SCD circumscribes a variety of conditions including pulmonary hypertension, pulmonary thromboembolism, asthma, and acute chest syndrome, most of which are discussed in this article [8]. This review article aims to cater a new and distinct perspective to the correlation between SCD and the lungs by (1) discussing the pathophysiological involvement of the lungs in SCD and (2) highlighting the screening guidelines and management of the above-mentioned diseases with respect to SCD.

## Review

Acute chest syndrome

Acute chest syndrome is known as a condition that is heralded by the onset of respiratory signs and symptoms like cough, wheezing, chest pain, fever, etc., along with the appearance of a lung infiltrate suggestive of consolidation on chest x-ray, in patients with SCD [9]. Carrying a high rate of morbidity and mortality, acute chest syndrome is one of the most important causes of hospitalizations in patients with SCD. There is growing evidence of the fact that more than half of the children suffering from the homozygous variant will be hospitalized at least once during the first decade of their life [10]. Resulting from a chaotic interplay between vasoocclusive ischemia and inflammatory cytokines, the lung undergoes a process of pathologic alveolar collapse leading to ventilation-perfusion mismatching, hypoxemia, and raised pulmonary pressures [11]. The sickled RBCs block the capillaries in the lungs resulting in decreased supply and exchange of oxygen. This further leads to the release of inflammatory mediators and adhesion molecules that cause the formation of minute clots in the blood vessel walls and hence, exacerbates the hypoxia [12].

**Figure 1:**
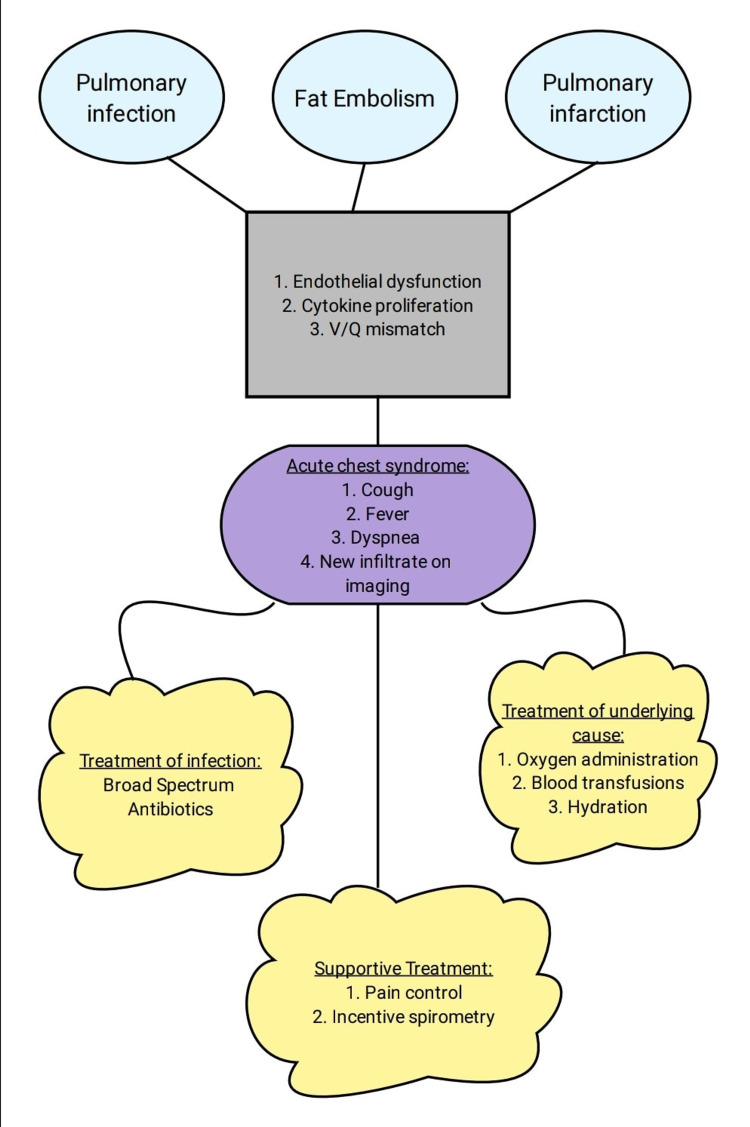
Summary of etiology, clinical features, and the management of acute chest syndrome. V/Q - Ventilation/Perfusion

The etiology of acute chest syndromes primarily revolves around three causes (Figure 1) [13]:

1. Pulmonary infection

2. Fat embolism

3. Pulmonary infarction

Pulmonary infections initiate a cascade of pro-inflammatory mediators and cytokines in the lung that leads to the sickling of RBCs [14]. Studies have shown an increase in the incidence of acute chest syndrome in patients with pulmonary infection with the causative agents ranging from *Chlamydia* *pneumoniae*, *Mycoplasma* *pneumoniae* to *Staphylococcus* *aureus* and *Streptococcus* ​​​​​*pneumoniae* [13].

Fat embolism is of peculiar importance in SCD as the necrosis of bone marrow during a vasoocclusive episode leads to the release of fat globules in the circulation, which then make their way up to the lungs, get metabolized by the enzymes locally, and precipitate proliferation of the inflammatory cytokines [15]. This leads to endothelial injury and an increase in the vascular permeability of the pulmonary tissue leading to acute chest syndrome [15].

A small percentage of cases of acute chest syndrome is brought upon by pulmonary infarction. Sickled RBCs obstructing the pulmonary circulation lead to a lack of blood flow and can cause infarction in some cases [13]. This ischemia induces cell injury in the surrounding tissues leading to hypoxemia and ventilation/perfusion (V/Q) mismatch in the affected parts of the lung [13].

The clinical presentation of acute chest syndrome usually varies by the age group of the patient. Children with age <10 have the highest incidence of acute chest syndrome most likely caused by pulmonary infection, whereas older children and adults tend to curve over with a more severe course with fat embolism being an important cause [16]. The common clinical features range between chest pain, wheezing, cough, and fever (Figure 1). However, adults can experience an increased occurrence of hypoxia, poor saturation, and basal lung involvement [13]. Due to the streamlined presentation, it should be noted that it can easily be confused with acute exacerbation of asthma, bronchiolitis, or bacterial pneumonia [16]. The course of illness is highly unpredictable and can progress from anywhere from mild hypoxemia to severe respiratory failure [13]. Incidence of neurological complications such as stroke and leukoencephalopathy was found to be higher in children who had experienced an episode of acute chest syndrome [17].

The diagnosis is predominantly clinical taking into consideration the patient's respiratory symptoms and radiological findings. Several biomarkers such as secretory phospholipase A2 (sPLA2), C-reactive protein (CRP), and pentraxin-related protein (PTX3) have often been correlated with the severity of the disease but they lack the sensitivity and specificity in the practical aspect of the diagnosis [15,18,19].

Initial diagnostic workup includes performing complete blood count, reticulocyte count, blood typing and cross-matching, blood cultures, and a chest X-ray [16]. This should be followed by immediate hospitalization for careful monitoring of the vitals, progression, and development of complications. The goals of the management include stabilizing adequate oxygen saturation (>94% in most cases), maintaining hydration by giving fluids, and preventing atelectasis by incentive spirometry, early mobilization, and chest exercises (Figure 1) [16]. Blood transfusions should be initiated to maintain hemoglobin levels above 9-11 g/dL or the concentration of HbS <30% [13]. An antibiotic regimen should be started by including broad-spectrum agents including coverage of methicillin-resistant *S. aureus* (MRSA) and atypical organisms and later tailoring it according to the blood culture reports (Figure 1) [13]. Pain control should also be offered to the patients including nonsteroidal anti-inflammatory drugs (NSAIDs) and opioids depending upon the severity to promote ventilation and prevent respiratory depression (Figure 1) [20]. Lastly, bronchodilators like albuterol should be given to improve hypoxemia [21].

Pulmonary hypertension in SCD

A person with SCD is at an elevated risk for developing pulmonary artery hypertension (PAH), which is known as a state where the resting pulmonary artery pressure exceeds 25 mm of Hg [22].

The Task Force for the Diagnosis and Treatment of Pulmonary Hypertension of the European Society of Cardiology and the European Respiratory Society published guidelines in 2009 to classify pulmonary hypertension along with the following types (Table 1) [23].

**Table 1:** Classification of Pulmonary Hypertension PAH - Pulmonary Artery Hypertension

Category	Cause
Group one	Idiopathic PAH
Group two	PAH secondary to left heart disease
Group three	PAH secondary to lung disease
Group four	PAH due to thromboembolism
Group five	PAH due to unknown etiology

Updated guidelines that were published in 2012, classified PAH due to SCD in Group five [24].

**Figure 2:**
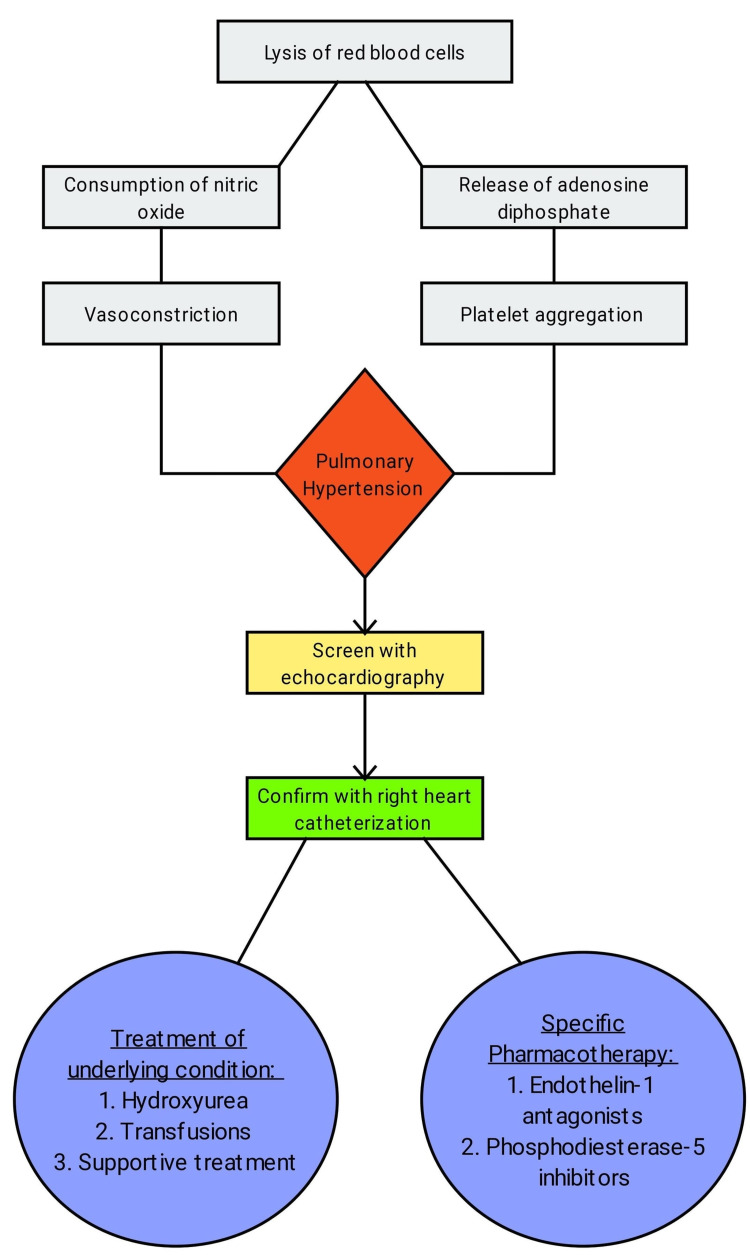
Summary of pathogenesis, investigations, and the management of pulmonary hypertension in sickle cell disease

Pulmonary hypertension in SCD results due to endothelial dysfunction secondary to an imbalance between vasoconstrictors and vasodilators [25]. When there is a lysis of RBCs in the circulation, the released hemoglobin consumes the nitric oxide (NO), which is a potent vasodilator (Figure 2) [26]. RBCs also release arginase on lysis, which causes breakdown and decreases the availability of arginine for the production of NO [26]. The lack of NO and release of adenosine diphosphate (ADP) by the lysed RBCs releases ADP that causes platelet activation. The formation of clots occludes the vessel, hampering the flow of blood through the vessel (Figure 2) [27]. Chronic vasoconstriction causes smooth muscle hypertrophy and endothelial proliferation and in turn, worsening the width of the vascular lumen to constricted flow and elevated pressure [28].

Although right heart catheterization is the gold standard for diagnosis of pulmonary hypertension, echocardiography is used to derive an estimate of the same using tricuspid regurgitation velocity (TRV) and right atrial pressure making it a useful screening tool (Figure 2) [29]. This does not imply the use of echocardiography in the diagnosis of pulmonary hypertension; rather helps in narrowing down the patient group that needs to undergo right heart catheterization. A TRV of <2.5 m/sec is usually considered to be normal [30]. Studies have found that TRV ranging between 2.5 and 2.8 m/sec in patients with SCD serves as a borderline indicator of pulmonary hypertension in <15% patients, whereas a TRV of >2.9 m/sec with a positive indicator of pulmonary hypertension in >50% of the patients [30]. The positively screened patients then undergo right heart catheterization to confirm the diagnosis [30].

The goals for the management of pulmonary hypertension in patients with SCD should be directed at minimizing the severity of the disease with Hydroxyurea therapy, improving the hemoglobin levels, and enhancing circulations by periodic blood transfusions, and targeted therapy towards decreasing pulmonary remodeling (Figure 2) [25]. Studies have shown that Sildenafil, a phosphodiesterase-5 (PDE-5) inhibitor led to the improvement in exercise tolerance in patients with pulmonary hypertension (Figure 2) [31]. Endothelin-1, a potent vasoconstrictor is found to be elevated in the lungs of patients with pulmonary hypertension due to SCD [32]. It leads to the dehydration of RBCs thus, promoting sickling. Endothelin-1 receptor antagonists such as Bosentan have also demonstrated efficacy in the treatment of pulmonary hypertension [33]. A noteworthy drug Epoprostenol, a short-acting potent vasodilator has portrayed effectiveness in increasing the six-minute walk distance and decreasing pulmonary vascular resistance in patients with pulmonary hypertension (Figure 2) [34].

Pulmonary thromboembolism

The fatality of pulmonary thromboembolism is increased in SCD because of the amplification of the parameters of the Virchows triad [35]. The sickled RBCs occlude the blood vessels during flow leading to endothelial injury, stasis, and increased expression of thrombogenic factors [35]. According to a study that was published in 2006 on African-Americans aged <40 years, it was concluded that patients suffering from SCD had an increased risk of pulmonary embolism compared to the control group [36]. The findings of the above study can be compared in parallel to another study that was performed in 2013, where it was found that people with TRV >2.5 m/s and variant subtypes had an elevated risk of developing thromboembolic events [37]. Some of the risk factors that increase the risk of pulmonary thromboembolism are mentioned in Table 2 [38].

**Table 2:** Summary of risk factors for thromboembolism

Risk factors for thromboembolism
1. Central venous catheters
2. Coexisting conditions like thalassemia
3. Surgery
4. Pregnancy
5. Splenectomy

There is an increased risk of incidence of the acute coronary syndrome and pulmonary hypertension that is directly proportional to the development of pulmonary thromboembolism [8]. As stated above, the group four category of the classification of pulmonary hypertension encompasses the etiologies that are caused by pulmonary thromboembolism. An interesting case report that was published in 2020 highlighted the presence of pulmonary hypertension due to thromboembolism in an 11-year-old child with a homozygous trait for SCD [38]. The use of parameters like D-dimers in patients with SCD for screening thromboembolism lacks reliability as the baseline D-dimer levels vary by a huge margin [39]. The management of thromboembolic events in patients with SCD revolves around individual factors like baseline hemoglobin values, the coexistence of other hematological abnormalities like thalassemia, and history of vasoocclusive/thromboembolic events. Like every other hypercoagulable disorder, the treatment generally involves the use of anticoagulants [40]. However, the benefit and risks of starting anticoagulation should be weighed against each other and tailored in with a planned chronic transfusion and hydroxyurea therapy [40].

Other pulmonary conditions

Apart from the conditions that are discussed above, other conditions such as asthma and sleep disorders have also been implicated in patients with SCD. 

Asthma and SCD, both of the conditions which rely heavily on the genetic inheritance of the patient, have demonstrated an increased rate of hospitalizations and mortality when present concurrently [41]. The diagnostic and prognostic question faced by pulmonologists and hematologists while managing the condition is that whether the presentation of cough, wheezing, and dyspnea is due to any of the two diseases individually or is an exacerbation of asthma as a result of SCD as there is a significant overlap in the symptomatology [42]. According to a study that was published in 2007, the forced vital capacity (FVC) and forced expiratory volume in one second (FEV1) were largely decreased on pulmonary function testing and the degree of airway hyper-responsiveness was increased on methacholine challenge test in children with SCD [43]. The treatment of asthma in sickle disease usually remains the same along the lines of allergen avoidance, medication adherence, beta 2 agonists, and corticosteroids [44].

Sleep-disordered breathing (SDB) is a common yet usually undiagnosed condition that leads to nocturnal hypoxemia in patients with SCD [45]. A retrospective review study done on children aged 2-18 years of age with a sample population of 55 revealed that 69% of the children (38/55) suffered from obstructive sleep apnea (OSA) concluding that there was an increased severity in patients with the homozygous trait [46]. The management of OSA in such cases is usually done by performing adenotonsillectomy, which leads to a reduction in the obstruction and thus, improving nocturnal hypoxemia [47].

Limitations

1. The respiratory conditions that have been reviewed in this article have a multifactorial web of etiology and usually involve more than a single causative factor such as SCD. This article focuses specifically on the effects of SCD that have been shadowed upon the respiratory system and ignores the other causes.

2. This study does not take into consideration the genetic vulnerability, environmental factors, and comorbidities of SCD patients that can potentially serve as a confounding variable. For example, the development of pulmonary hypertension in patients could have also been brought upon by non-modifiable and modifiable factors like genetic mutations and smoking, respectively.

## Conclusions

As evident from the studies reviewed in this article that despite having a predominant hematological involvement, the disease is also a causative factor for a complex multi-system involvement. In summary, the clinical implication of this review article is to establish a strong link between SCD and respiratory conditions such as acute chest syndrome, pulmonary hypertension, pulmonary thromboembolism, asthma, and sleep disorders. These conditions are strongly associated with amplified risk of mortality, decreased quality of life, and increased rates of hospitalizations. We believe that this article can serve as a tool to overcome these challenges by providing a unique approach to the connection between the two entities by highlighting the pathogenesis, clinical manifestations, and management options. We spoke specifically of challenges that are faced by physicians while tackling the miscellaneous association between SCD and the respiratory system due to a delay in diagnosis, overlap in the presenting symptomatology, and complex multicentric management. Furthermore, these obstacles could also be overcome by a disciplined treatment regimen for SCD engineered individually according to patient factors and close monitoring of the hematological and respiratory parameters. Lastly, we feel that the association between SCD and the respiratory system needs more in-depth research studies to be performed to construct a more organized and direct approach to diagnose and manage these conditions.
